# Robotic-assisted versus conventional coronary artery bypass grafting: a systematic review, meta-analysis, and trial sequential analysis of 39,918 patients

**DOI:** 10.1186/s13019-026-04684-8

**Published:** 2026-10-12

**Authors:** Ahmed Abdelsamad, Ibrahim Khalil, Mohammed Hrebat, Taher Abuawwad, Momen Zetawi, Basem Mohamed Yassin, Samer Imad Amayre, Mahmoud Ali Khazbak, Nadjet Laouadi, Shaimaa Khatib, Hamza Motaz Atalla, Layth Adnan Manasra, Abdellatif Qaraqe, Baraa Mansour, Tarig Mohamed Abdelatief Abdeldaim, Tamer Ghazy

**Affiliations:** 1 Department of Surgery II, University of Witten/Herdecke, Witten, Germany; 2 Chief, General and Gastrointestinal surgery, Helios Clinic Cuxhaven, Altenwalder Chaussee 10-12, 27474, Cuxhaven, , Germany; 3 Faculty of Medicine, Alexandria University, Alexandria, Egypt; 4 Faculty of Medicine, Tanta University, Tanta, Egypt; 5 Fujian Medical University, Fujian, China; 6 Faculty of Medicine, An-Najah National University, Nablus, Palestine; 7 Faculty of Medicine, Kuwait University, Kuwait City, Kuwait; 8 Faculty of Medicine, Al-Quds University, Jerusalem, Palestine; 9 Ministry of Health, Muscat, Oman; 10 Heart Center, King Abdulaziz Specialized Hospital, Al Jouf, Saudi Arabia; 11 Faculty of Medicine, Cairo University, Cairo, Egypt; 12 Faculty of Medicine, Ain Shams University, Cairo, Egypt; 13 Omdurman Islamic University, Omdurman, Sudan; 14 Department of Cardiac Surgery, Philipps University of Marburg, Marburg, Germany

**Keywords:** Robotic surgery, Coronary artery bypass grafting, CABG, Meta-analysis, Trial sequential analysis, Minimally invasive cardiac surgery, TECAB

## Abstract

**Background:**

Robotic-assisted coronary artery bypass grafting (RA-CABG) is an evolving minimally invasive alternative to conventional CABG (C-CABG), but comparative evidence remains fragmented. We conducted a comprehensive systematic review, meta-analysis, and trial sequential analysis to compare outcomes between RA-CABG and C-CABG.

**Methods:**

Following PRISMA 2020 guidelines, we searched PubMed, Embase, Cochrane CENTRAL, and Web of Science through January 2026. Studies comparing RA-CABG to C-CABG reporting at least one outcome of interest were eligible. Random-effects meta-analyses using DerSimonian–Laird estimators were performed. Risk of bias was assessed using ROBINS-I. Trial sequential analysis evaluated the conclusiveness of cumulative evidence.

**Results:**

Nine studies comprising 39,918 patients (13,638 RA-CABG; 26,280 C-CABG) were included. All included studies were observational in design, and long-term outcome data were sparse. RA-CABG was associated with significantly lower mortality (OR 0.55, 95% CI 0.43–0.69; *P* < 0.001; I²=0%), atrial fibrillation (OR 0.53; *P* < 0.001), respiratory failure (OR 0.31; *P* < 0.001), blood transfusion (OR 0.28; *P* < 0.001), hospital length of stay (MD − 1.95 days; *P* < 0.001), and ICU stay (MD − 22.7 h; *P* < 0.001). Stroke showed a non-significant trend favoring RA-CABG (OR 0.76; *P* = 0.071). Trial sequential analysis confirmed conclusive evidence for mortality, atrial fibrillation, respiratory failure, and blood transfusion. Results were robust across sensitivity analyses.

**Conclusions:**

In this meta-analysis of observational studies, RA-CABG was associated with significantly improved short-term outcomes compared to C-CABG across multiple endpoints, with conclusive statistical evidence for four of seven binary outcomes. However, all included studies were observational, and these associations should be interpreted with caution given potential residual confounding and case-selection bias. Multicenter randomized trials with long-term follow-up are needed to confirm these findings.

**Supplementary Information:**

The online version contains supplementary material available at https://doi.org/10.1186/s13019-026-04684-8.

## Introduction

 Coronary artery disease (CAD) remains the leading cause of mortality worldwide, accounting for approximately 9.14 million deaths annually [1]. Coronary artery bypass grafting (CABG) is the gold-standard revascularization strategy for patients with multivessel or left main coronary disease, as endorsed by the American College of Cardiology/American Heart Association (ACC/AHA) [2] and the European Society of Cardiology (ESC) [3]. Despite its proven efficacy, conventional CABG via median sternotomy is associated with significant morbidity, including sternal wound complications [4], prolonged recovery, and postoperative pain that can limit rehabilitation and return to functional independence [5].

The advent of robotic surgical systems has revolutionized multiple surgical disciplines, offering enhanced visualization, improved dexterity, and the potential for minimally invasive approaches [6]. In cardiac surgery, robotic-assisted CABG (RA-CABG) was first reported by Loulmet and Carpentier in 1999 [7], and the technique has since evolved to enable totally endoscopic coronary artery bypass (TECAB) through small port incisions using the da Vinci Surgical System, potentially eliminating the need for sternotomy and cardiopulmonary bypass in select cases [8]. Importantly, robotic-assisted CABG encompasses a spectrum of techniques with varying degrees of invasiveness, including totally endoscopic CABG (TECAB), robotic-assisted minimally invasive direct coronary artery bypass (RA-MIDCAB), and robotic-assisted mini-CABG via small thoracotomy, each with distinct technical demands and applicability to different patient populations.

Several single-center studies and registry analyses have reported promising outcomes with RA-CABG, including reduced wound complications, shorter hospital stays, and faster return to normal activities [9, 10]. However, concerns persist regarding longer operative times, steep learning curves, and the applicability of the technique to patients requiring multivessel revascularization [10, 11]. Furthermore, the high capital and operational costs of robotic systems raise questions about cost-effectiveness in resource-limited settings [11].

Previous systematic reviews have noted that the overall quality of the evidence was low, and in the absence of randomized controlled trials, results should be interpreted cautiously [12]. Recent publication of large national database studies and contemporary matched cohort analyses provides an opportunity for more comprehensive analysis. Therefore, we conducted a systematic review and meta-analysis to compare the outcomes of RA-CABG versus C-CABG, encompassing mortality, morbidity, and resource utilization across multiple endpoints.

## Methods

### Protocol and registration

This systematic review was conducted in accordance with the Preferred Reporting Items for Systematic Reviews and Meta-Analyses (PRISMA) 2020 guidelines [13]. The protocol was prospectively registered in PROSPERO (CRD420251250311).

### Search strategy

A comprehensive literature search was performed across PubMed/MEDLINE, Embase, Cochrane CENTRAL, and Web of Science from inception through January 31, 2026. The search strategy combined Medical Subject Headings (MeSH) terms and free-text keywords related to robotic surgery and coronary artery bypass grafting. The complete search strategy is provided in Supplementary Table S1. Reference lists of included studies and relevant review articles were manually screened.

### Eligibility criteria

Studies were included if they: (1) compared robotic-assisted CABG to conventional CABG; (2) reported at least one outcome of interest; (3) included adult patients (≥ 18 years); and (4) were published as full-text articles. Exclusion criteria included: conference abstracts without full publication, duplicate patient cohorts, mixed populations without separate CABG data, studies combining robotic with other minimally invasive techniques without separate reporting, and pediatric populations.

### Study selection and data extraction

Two reviewers independently screened titles and abstracts, followed by full-text review of potentially eligible studies. Disagreements were resolved by consensus or consultation with a third reviewer. When multiple publications from the same institution or database were identified, the study with the largest cohort and most comprehensive outcome reporting was selected to avoid duplicate patient inclusion; excluded overlapping publications are documented in the PRISMA flow diagram under ‘duplicate patient cohort (n = 6).’ Data extraction was performed using a standardized form capturing study characteristics, patient demographics, surgical details, and outcomes. Double data extraction was performed independently by two reviewers for all primary and secondary outcomes; discrepancies were resolved by re-verification against the source publications. Every extracted value was verified line-by-line against the original full-text publications, with exact source table and line numbers documented in a formal audit trail. Studies reporting medians with interquartile ranges had means and standard deviations estimated using the method described by Wan et al. [14]; this method was applied to hospital length of stay and ICU stay where studies reported medians. Where raw event counts were unavailable due to entropy-balanced or regression-adjusted designs (Verma 2025: adjusted OR for mortality; Liu 2025: Cox HR for stroke), adjusted estimates were incorporated using generic inverse-variance methods and reported separately from pooled binary analyses. Unadjusted raw event counts were used for standard pooling; adjusted estimates were incorporated only when raw counts were unavailable or when study design precluded standard pooling, and these are clearly identified throughout the Results.

### Outcomes

Primary outcomes were in-hospital or 30-day mortality and stroke. Secondary outcomes included new-onset atrial fibrillation, myocardial infarction, renal failure, respiratory failure or prolonged mechanical ventilation (> 24 h), sepsis, deep sternal wound infection (DSWI), blood transfusion, reoperation for bleeding, length of hospital stay (LOS), and intensive care unit (ICU) stay.

### Risk of bias assessment

Risk of bias was assessed using the Risk of Bias in Non-randomized Studies of Interventions (ROBINS-I) tool [15]. Domains evaluated included confounding, selection of participants, classification of interventions, deviations from intended interventions, missing data, measurement of outcomes, and selection of reported results. Overall risk was classified as low, moderate, or serious.

### Statistical analysis

Meta-analyses were conducted using random-effects models with the DerSimonian–Laird estimator [16] to account for anticipated clinical and methodological heterogeneity. Effect estimates were expressed as odds ratios (ORs) with 95% confidence intervals (CIs) for binary outcomes and mean differences (MD) for continuous outcomes. A continuity correction of 0.5 was applied to studies with zero events in one arm. Heterogeneity was evaluated using Cochran’s Q test and the I² statistic, with I² values interpreted as low (0–25%), moderate (25–50%), substantial (50–75%), or considerable (> 75%), as described by Higgins et al. [17].

Verma 2025, which used entropy balancing with substantially unequal groups (3,433 vs. 18,437), was handled separately: its adjusted OR for mortality (AOR 0.35, 95% CI 0.15–0.84) was incorporated via generic inverse-variance and reported as a confirmatory analysis. Raw event counts from entropy-balanced unequal groups were not pooled in standard forest plots. Liu 2025, which reported stroke exclusively as a Kaplan–Meier hazard ratio (HR 0.771, 95% CI 0.600–0.991), was incorporated descriptively and not pooled in the binary stroke forest plot.

Drexel 2016, an unmatched retrospective comparison with extreme baseline imbalance (3-vessel CAD: 26% robotic vs. 78% conventional), was rated Serious risk of bias on ROBINS-I and included in sensitivity analysis only.

Sensitivity analyses included leave-one-out analyses and pre-planned exclusion of the unmatched cohort (Drexel 2016). Prespecified subgroup analyses were performed according to publication year (< 2020 vs. ≥ 2020), geographic region (North America vs. Europe), and study size (< 500 vs. ≥ 500 patients). Meta-regression using mixed-effects models explored publication year as a potential source of heterogeneity. Publication bias was evaluated by funnel plots and Egger’s regression test for outcomes with sufficient studies. All analyses were performed in R (version 4.3.3) using the meta (v8.2) and metafor (v4.8) packages, with two-sided *P* < 0.05 considered statistically significant.

### Trial sequential analysis

Trial sequential analysis (TSA) was performed to evaluate whether the cumulative evidence for each binary outcome had reached sufficient statistical reliability, accounting for the risk of random error due to sparse data and repetitive significance testing across sequentially published studies. For each outcome, the required information size (RIS) was calculated assuming a two-sided type I error of 5%, a power of 80%, and a relative risk reduction (RRR) of 40% based on the observed control event rate. The O’Brien-Fleming alpha-spending function was applied to construct sequential monitoring boundaries. Studies were added chronologically by publication year, and the cumulative Z-statistic was plotted against accrued sample size. An outcome was deemed conclusive if the Z-curve crossed the TSA monitoring boundary; sufficient but subthreshold if the accrued information exceeded the RIS but the Z-curve did not cross the monitoring boundary (indicating the observed effect size is smaller than the assumed RRR); inconclusive if the Z-curve remained within the boundaries without reaching the RIS; and underpowered if the accrued information was substantially below the RIS. TSA was performed using R (version 4.3.3) with the metafor package.

## Results

### Study selection

**Fig. 1:**
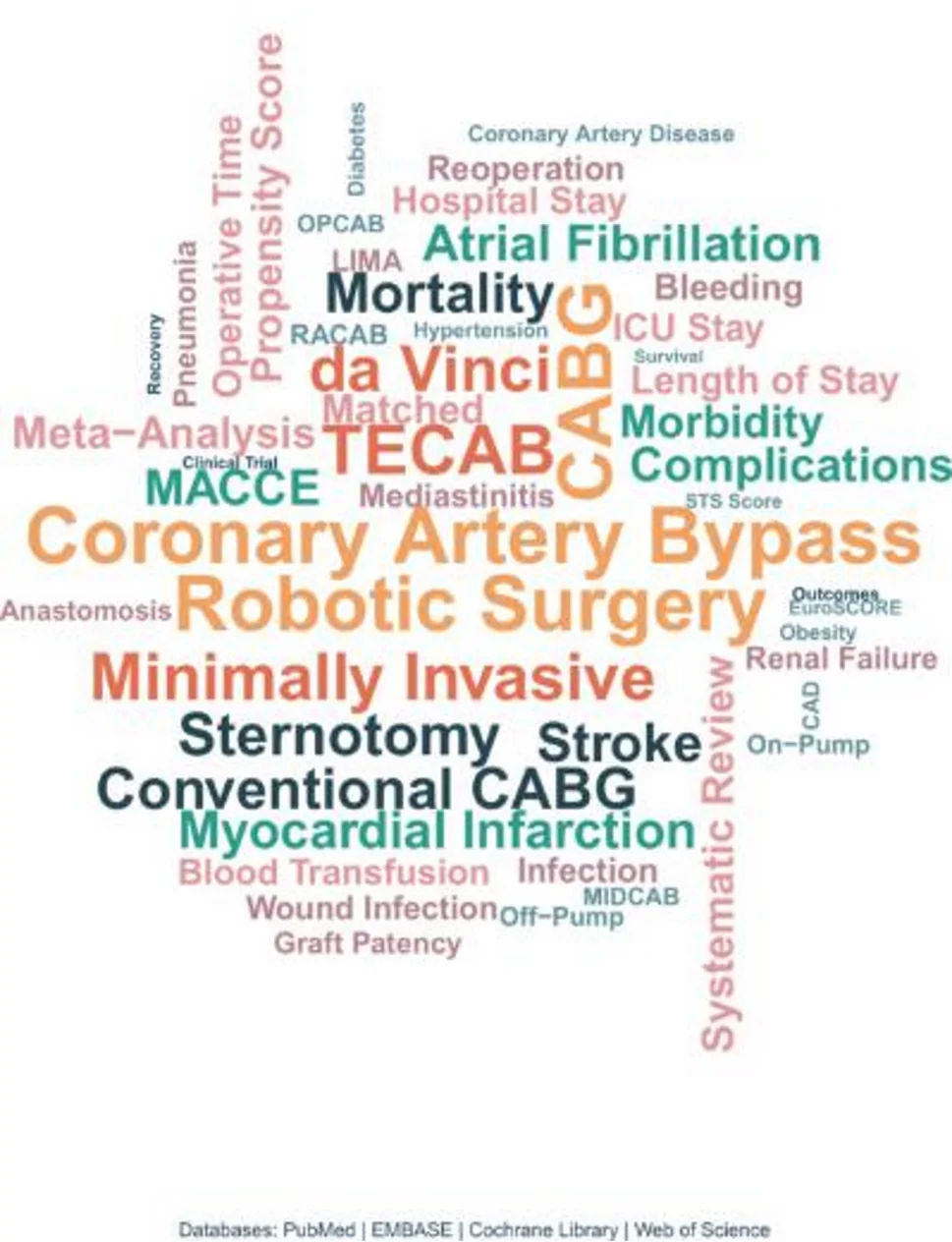
Word Cloud Visualization of Key Terms in Robotic-Assisted CABG Literature

The systematic search identified 1,847 records across all databases. After removing duplicates, 1,612 records were screened by title and abstract, of which 1,565 were excluded. Full-text review of 47 articles led to exclusion of 38 studies for the following reasons: not comparative study design (*n* = 12), conference abstract only without full publication (*n* = 8), duplicate patient cohort (*n* = 6), mixed population without separate CABG data (*n* = 5), hybrid procedures only without separate robotic reporting (*n* = 4), insufficient outcome data (*n* = 2), and pediatric population (*n* = 1). Nine studies met the inclusion criteria and were included in the meta-analysis (Fig. 1).

### Study characteristics

The nine included studies [S1–S9] comprised 39,918 patients: 13,638 (34.2%) in the RA-CABG group and 26,280 (65.8%) in the C-CABG group. Studies were published between 2008 and 2025. Geographic distribution included North America (*n* = 7, all USA), Europe (*n* = 1, Austria), and one international multicenter study using the TriNetX platform. Study designs included seven propensity score-matched analyses, one entropy-balanced retrospective study using the Nationwide Readmissions Database, and one unmatched retrospective comparison (included in sensitivity analysis only due to serious risk of bias). Sample sizes ranged from 200 to 21,870 patients per study. Baseline characteristics of included studies are presented in Table 1.

**Table 1:** Characteristics of Included Studies

Study	Country	Period	Design	Matching	*N* (RA)	*N* (C)	Age RA (yr)	Age C (yr)	Male (%RA/%C)	DM (%RA/%C)	Grafts (RA/C)	Journal
Kofler 2017	Austria	2001–2013	RC	PSM	134	134	62 ± 10	62 ± 10	NR	NR	1.3/1.6	Innovations
Poston 2008	USA	2005–2007	RC	PSM	100	100	61.8 ± 9.4	66.2 ± 10.1	72/63	32/43	1.9/2.9	Ann Surg
Leyvi 2015	USA	2007–2012	RC	PSM	141	141	64.7 ± 12.8	64.4 ± 9.9	67/70	50/48	1.1/3.1	JCVA
Drexel 2016*	USA	2003–2014	RC	None	99	300	65 ± 11	63 ± 11	68/73	42/47	1.7/3.5	J Cardiol Cardiovasc Ther
Yokoyama 2021	USA	2012–2017	NDB	PSM	7,330	7,330	65.0 ± 10.9	65.4 ± 10.7	74/74	41/38	NR	J Card Surg
Dokollari 2023	USA	2018–2021	RC	PSM	267	267	69.5 ± 10.3	70.1 ± 8.4	78/75	46/46	1.1/2.9	Am J Cardiol
Liu 2025	Int’l†	2005–2025	NDB	PSM	1,796	1,796	64.9 ± 11.2	65.1 ± 10.9	76/76	31/30	NR	J Robot Surg
Zhou 2025	USA	2014–2023	RC	PSM	338	338	NR‡	NR‡	NR‡	NR‡	~ 1.8/~2.9	Ann Thorac Surg Short Rep
Verma 2025	USA	2016–2020	NDB	EB§	3,433	18,437	65.5 ± 11.4	65.0 ± 10.5	79/70	NR	1 V only	Ann Thorac Surg Short Rep
**TOTAL (9 studies)**					**13**,**638**	**26**,**280**						

RC = retrospective cohort; NDB = national database study; PSM = propensity score matching; EB = entropy balancing; NR = not reported

*Drexel 2016: Unmatched design (serious risk of bias); included in sensitivity analysis only

†Liu 2025: TriNetX multinational federated database

‡Zhou 2025: Matched on age, sex, STS-PROM, BMI; exact values not reported in publication

§Verma 2025: NRD 2016–2020; entropy-balanced weighting (single-vessel CABG only); raw event counts not poolable

Robotic techniques included totally endoscopic coronary artery bypass (TECAB: Kofler 2017), robotic-assisted minimally invasive direct coronary artery bypass (RMIDCAB: Zhou 2025), robotic-assisted mini-CABG (Poston 2008), and unspecified robotic-assisted CABG using the da Vinci Surgical System (remaining studies). The number of grafts performed was consistently lower in RA-CABG cohorts (range 1.1–1.9) compared to C-CABG cohorts (range 2.5–3.1), consistent with the predominant application of RA-CABG for single-vessel or limited multivessel disease. Among studies reporting coronary disease extent, RA-CABG patients predominantly had single-vessel or double-vessel disease, whereas C-CABG patients more frequently had triple-vessel disease (e.g., Drexel 2016: 3-vessel CAD 26% robotic vs. 78% conventional). None of the included studies systematically reported rates of concomitant or staged percutaneous coronary intervention (PCI) to non-LAD vessels (hybrid revascularization) alongside robotic CABG.

### Risk of bias assessment

**Fig. 2:**
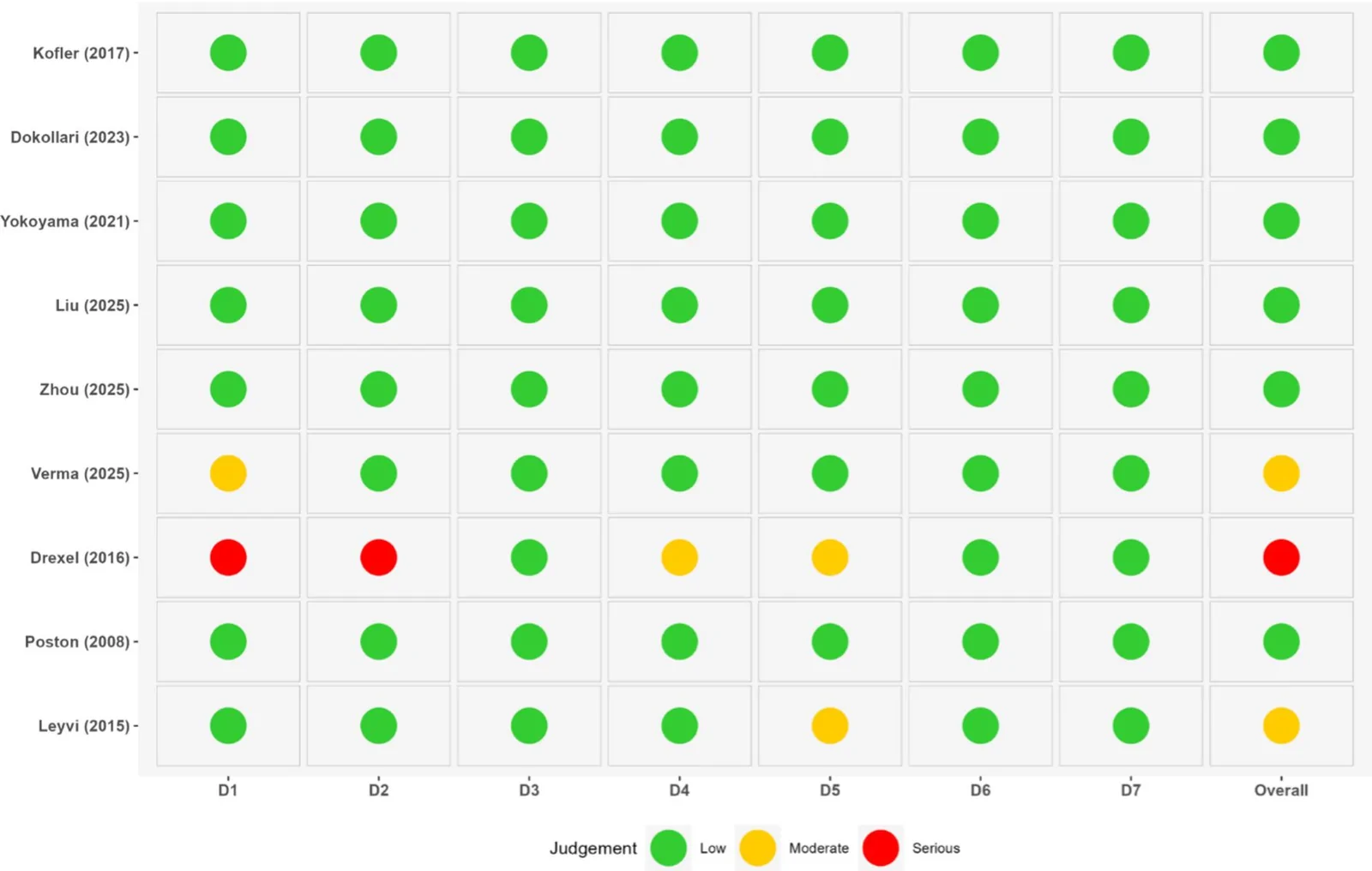
ROBINS-I Risk of Bias Assessment — Traffic Light Plot

Risk of bias assessment using ROBINS-I revealed six studies at low overall risk (Kofler 2017, Dokollari 2023, Yokoyama 2021, Liu 2025, Zhou 2025, Poston 2008), two at moderate risk (Verma 2025: entropy balancing with substantially unequal groups; Leyvi 2015: potential missing data in secondary outcomes), and one at serious risk (Drexel 2016: no matching with extreme baseline imbalance in 3-vessel CAD rates, 26% vs. 78%). The most common source of potential bias was confounding, although seven of nine studies employed propensity score matching or entropy balancing. Detailed assessments are presented in Fig. 2 and Supplementary Figure S1.

### Overview of meta-analysis results

RA-CABG was associated with significantly improved outcomes across the majority of endpoints evaluated. Results are presented below according to primary outcomes (mortality and stroke), secondary binary outcomes, and resource utilization outcomes. A summary of all pooled results is presented in Table 2.

**Table 2:** Summary of Pooled Meta-Analysis Results

Outcome	Studies (*N*)	Robotic Events/Total	Conventional Events/Total	Pooled OR or MD (95% CI)	*p*-value	I²	Figure	Interpretation
**Primary outcomes**								
Mortality (30-day/in-hosp)	8	108/10,245	202/10,445	OR 0.55 (0.43–0.69)	< 0.001	0%	Figure 3	Significant ↓ Robotic
Stroke	7	79/8,543	107/8,543	OR 0.76 (0.57–1.02)	0.071	0%	Figure 4	NS (trend ↓ Robotic)
**Secondary binary outcomes**								
Atrial Fibrillation (new)	4	141/804	283/1,005	OR 0.53 (0.42–0.66)	< 0.001	0%	Figure 5	Significant ↓ Robotic
Renal Failure	5	65/8,176	74/8,176	OR 0.73 (0.46–1.16)	0.184	0%	Supp. Fig. S3	NS
Respiratory Failure / PV	4	20/846	62/846	OR 0.31 (0.18–0.52)	< 0.001	0%	Figure 6	Significant ↓ Robotic
Reoperation for Bleeding	4	13/846	15/846	OR 0.82 (0.21–3.11)	0.767	54%	Supp. Fig. S4	NS
Blood Transfusion	3	865/7,738	1,964/7,738	OR 0.28 (0.17–0.45)	< 0.001	85%	Figure 7	Significant ↓ Robotic
Sepsis	3	81/7,809	147/7,809	OR 0.29 (0.04–2.30)	0.240	60%	Supp. Fig. S5	NS (underpowered)
**Recovery outcomes (Continuous)**								
Hospital LOS (days)	6	*N* = 8,275	*N* = 15,976	MD − 1.95 (− 2.30 to − 1.60)	< 0.001	54%	Figure 8	Significant ↓ Robotic
ICU Stay (hours)	5	*N* = 945	*N* = 1,246	MD − 22.7 (− 31.4 to − 14.0)	< 0.001	83%	Figure 9	Significant ↓ Robotic

OR = Odds Ratio; MD = Mean Difference; CI = Confidence Interval; I² = heterogeneity statistic; NS = Not Statistically Significant; PV = Prolonged Ventilation (> 24 h); LOS = Length of Stay; ICU = Intensive Care Unit. All analyses used random-effects models (DerSimonian-Laird). ↓ Robotic = outcome favoring robotic-assisted CABG

**Table 3:**
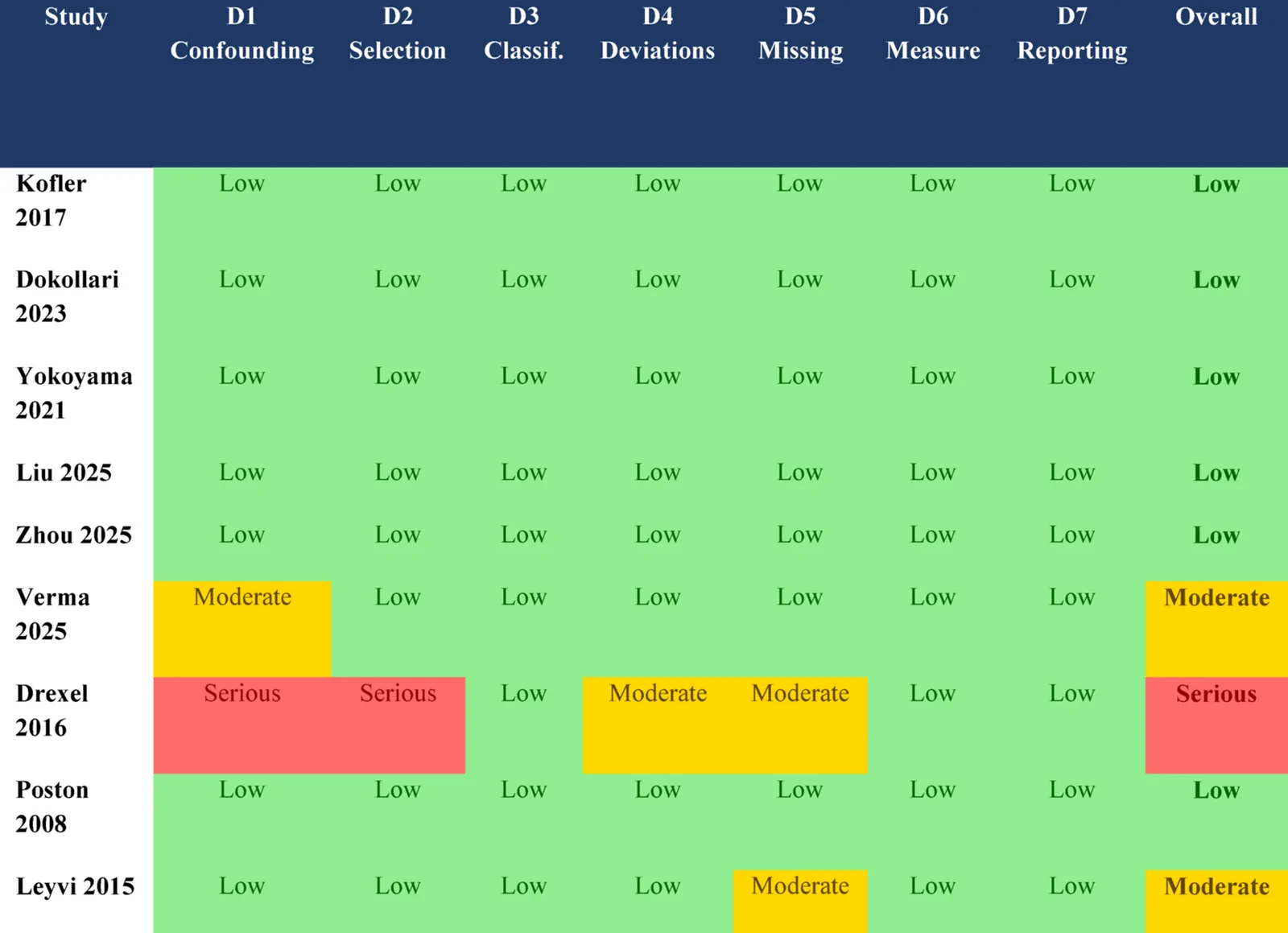
ROBINS-I Risk of Bias Assessment

*D1:Confounding; D2: Selection of participants; D3: Classification of interventions; D4: Deviations from intended interventions; D5: Missing outcome data; D6: Measurement of outcomes; D7: Selection of reported results. Color coding: Green = Low; Yellow = Moderate; Red = Serious risk of bias*

**Table 4:** GRADE Summary of Findings — RA-CABG vs. C-CABG

Outcome	No. of Studies (k)	Patients (Total)	Effect Estimate (95% CI)	I²	Risk of Bias	Inconsistency	Indirectness	Imprecision	Publication Bias	Overall Certainty
Mortality (30d/In-hosp)	8	20,690	OR 0.55 (0.43–0.69)	0%	Not serious	Not serious	Not serious	Not serious	Undetected	⊕⊕⊕◯ Moderate¹
Stroke	7	17,086	OR 0.76 (0.57–1.02)	0%	Not serious	Not serious	Not serious	Serious²	Undetected	⊕⊕◯◯ Low¹ˈ²
Atrial Fibrillation	4	1,809	OR 0.53 (0.42–0.66)	0%	Not serious	Not serious	Not serious	Not serious	N/A	⊕⊕⊕◯ Moderate¹
Resp Failure	4	1,692	OR 0.31 (0.18–0.52)	0%	Not serious	Not serious	Not serious	Not serious	N/A	⊕⊕⊕◯ Moderate¹
Blood Transfusion	3	15,476	OR 0.28 (0.17–0.45)	85%	Not serious	Serious³	Not serious	Not serious	N/A	⊕⊕◯◯ Low¹ˈ³
Renal Failure	5	16,352	OR 0.73 (0.46–1.16)	0%	Not serious	Not serious	Not serious	Serious²	Undetected	⊕⊕◯◯ Low¹ˈ²
Reoperation Bleeding	4	1,692	OR 0.82 (0.21–3.11)	54%	Not serious	Serious³	Not serious	Serious²	N/A	⊕◯◯◯ Very Low¹ˈ²ˈ³
Hospital LOS (days)	6	24,251	MD − 1.95 (− 2.30, − 1.60)	54%	Not serious	Serious³	Not serious	Not serious	Undetected	⊕⊕◯◯ Low¹ˈ³
ICU LOS (hours)	5	2,191	MD − 22.7 (− 31.4, − 14.0)	83%	Not serious	Serious³	Not serious	Not serious	N/A	⊕⊕◯◯ Low¹ˈ³

¹ Downgraded one level: all studies are observational

² Downgraded one level: wide CI crossing null (imprecision)

³ Downgraded one level: substantial heterogeneity (I² > 50%)

GRADE ratings: ⊕⊕⊕⊕ High; ⊕⊕⊕◯ Moderate; ⊕⊕◯◯ Low; ⊕◯◯◯ Very Low

N/A = fewer than 10 studies

† Effect estimates corrected in R2 to match primary pooled analysis (Table 2)

**GRADE Certainty of Evidence**

The certainty of evidence was assessed using the Grading of Recommendations Assessment, Development and Evaluation (GRADE) framework (Table 4). All included studies were observational in design, resulting in an initial rating of low certainty for all outcomes. For mortality, atrial fibrillation, and respiratory failure, the evidence was upgraded to moderate certainty due to a large and consistent magnitude of effect (OR < 0.55), minimal heterogeneity (I² = 0%), narrow confidence intervals, and concordant results across leave-one-out sensitivity and prespecified subgroup analyses. Stroke and renal failure were rated as low certainty due to imprecision, with confidence intervals crossing the null. Blood transfusion, hospital length of stay, and ICU length of stay were rated as low certainty owing to substantial heterogeneity (I² > 50%). Reoperation for bleeding was rated as very low certainty due to both imprecision and inconsistency (I² = 54%). No outcome was rated as high certainty, consistent with the inherent limitations of non-randomized evidence

### Primary outcomes

#### Mortality (30-Day / In-Hospital)

**Fig. 3:**
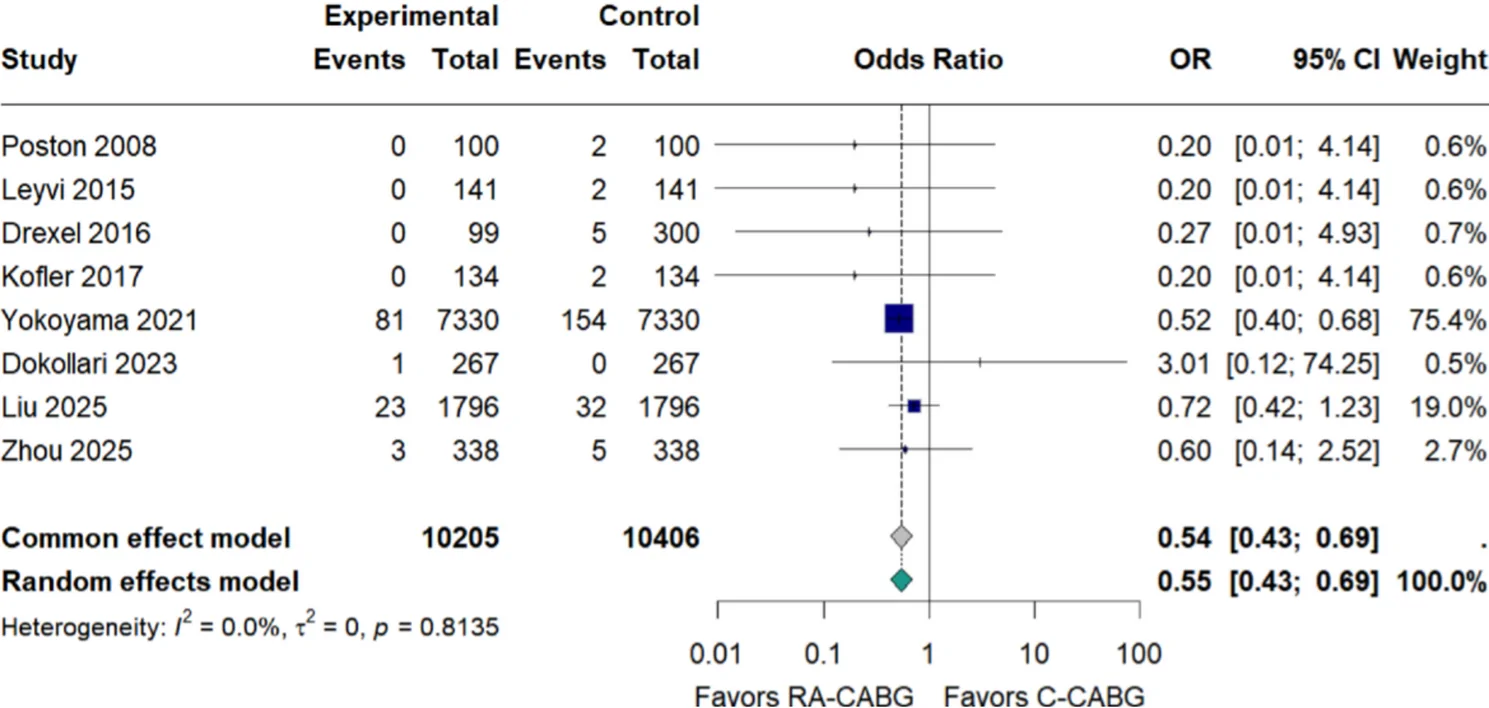
Forest Plot — Mortality (30-Day / In-Hospital)

Eight studies with extractable event counts reported short-term mortality (30-day or in-hospital). RA-CABG was associated with significantly lower mortality compared to C-CABG (OR 0.55, 95% CI 0.43–0.69; *P* < 0.001, I² = 0%; 108/10,245 [1.05%] vs. 202/10,445 [1.93%]; Fig. 3). This corresponds to an absolute risk difference of 0.88% points (number needed to treat [NNT] = 114). The direction of effect was consistent across all eight contributing studies; no single study drove the result on leave-one-out analysis (Supplementary Figure S2). Results remained robust in sensitivity analysis excluding the unmatched Drexel 2016 cohort (Supplementary Figure S8) Tables 3, 4, 5.

Additionally, Verma 2025—the largest cohort in this review (21,870 patients)—independently reported an adjusted OR of 0.35 (95% CI 0.15–0.84; *P* = 0.019) for in-hospital mortality after entropy balancing. Because this study used unequal groups (3,433 robotic vs. 18,437 conventional) and a non-standard balancing method, its adjusted estimate was incorporated via generic inverse-variance and is reported separately as a confirmatory analysis rather than pooled with raw event counts. This finding further supports the association between RA-CABG and lower short-term mortality.

#### Stroke

**Fig. 4:**
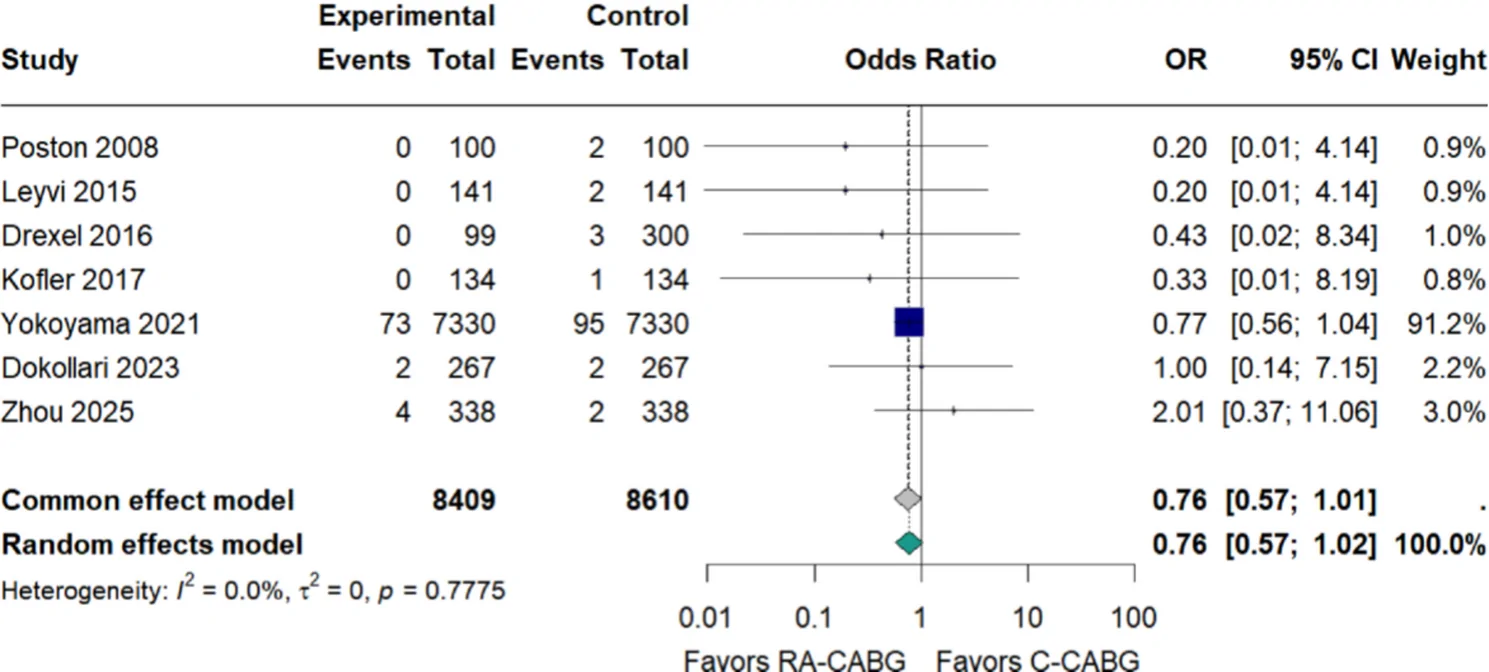
Forest Plot — Stroke

Seven studies with extractable event counts reported stroke. The pooled analysis did not demonstrate a statistically significant difference between RA-CABG and C-CABG (OR 0.76, 95% CI 0.57–1.02; *P* = 0.071, I² = 0%; 79/8,543 [0.92%] vs. 107/8,543 [1.25%]; Fig. 4). This corresponds to an absolute risk difference of 0.33% points. Although the point estimate favored RA-CABG, the 95% confidence interval crossed 1.0.

Liu 2025, which reported stroke as a Kaplan–Meier hazard ratio with median follow-up of 49 months—the longest follow-up in this review—found a significant reduction in cerebrovascular events associated with RA-CABG (HR 0.771, 95% CI 0.600–0.991; *P* = 0.042). As this was the only study reporting stroke as a time-to-event outcome, the hazard ratio was not pooled with the binary event counts but is reported separately. This long-term result suggests a possible sustained cerebrovascular association that short-term pooled analyses may not capture.

### Secondary binary outcomes

#### Atrial fibrillation

**Fig. 5:**
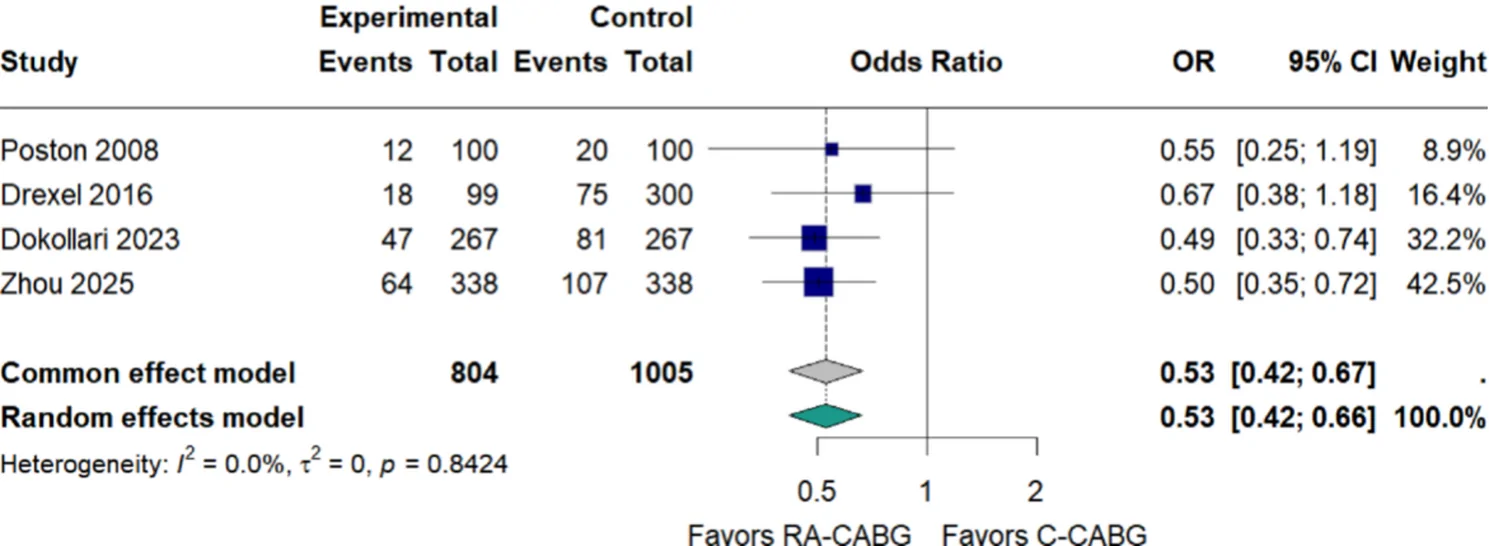
Forest Plot — Atrial Fibrillation (New-Onset)

Four studies reported new-onset atrial fibrillation. RA-CABG was associated with significantly lower rates compared to C-CABG (OR 0.53, 95% CI 0.42–0.66; *P* < 0.001, I² = 0%; 141/804 [17.5%] vs. 283/1,005 [28.2%]; Fig. 5).

#### Respiratory failure / prolonged ventilation

**Fig. 6:**
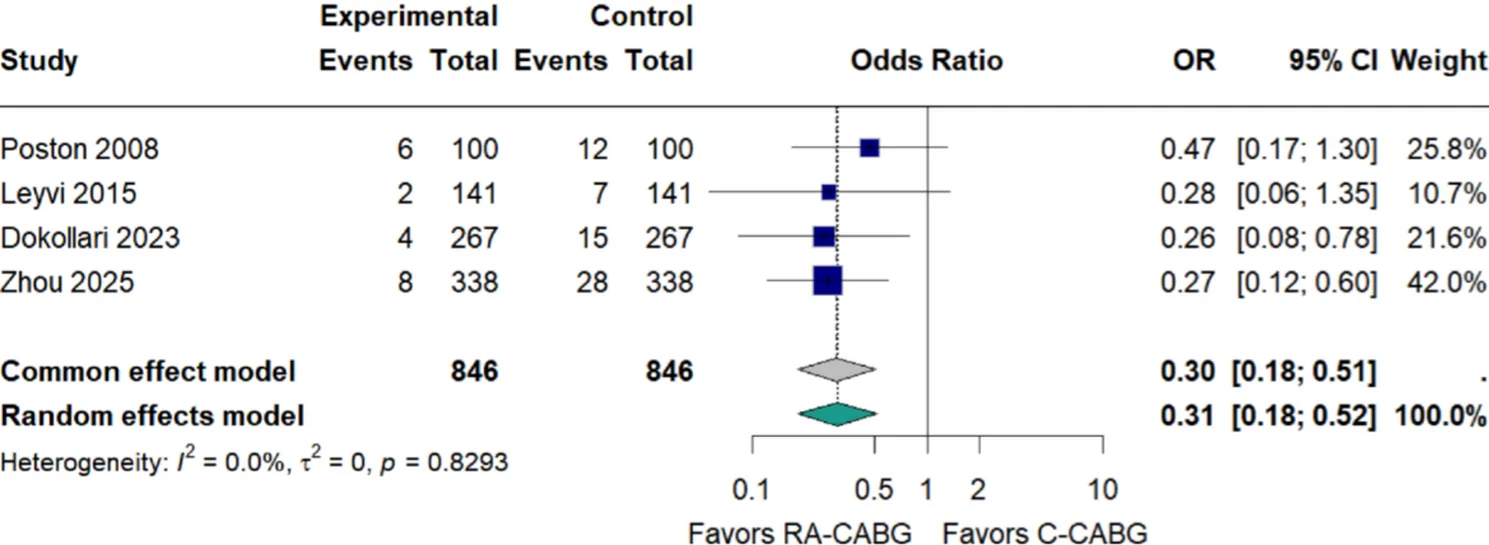
Forest Plot — Respiratory Failure / Prolonged Ventilation

Four studies reported postoperative respiratory failure or prolonged mechanical ventilation (> 24 h). RA-CABG was associated with markedly lower rates compared to C-CABG (OR 0.31, 95% CI 0.18–0.52; *P* < 0.001, I² = 0%; 20/846 [2.4%] vs. 62/846 [7.3%]; Fig. 6).

#### Blood transfusion

**Fig. 7:**
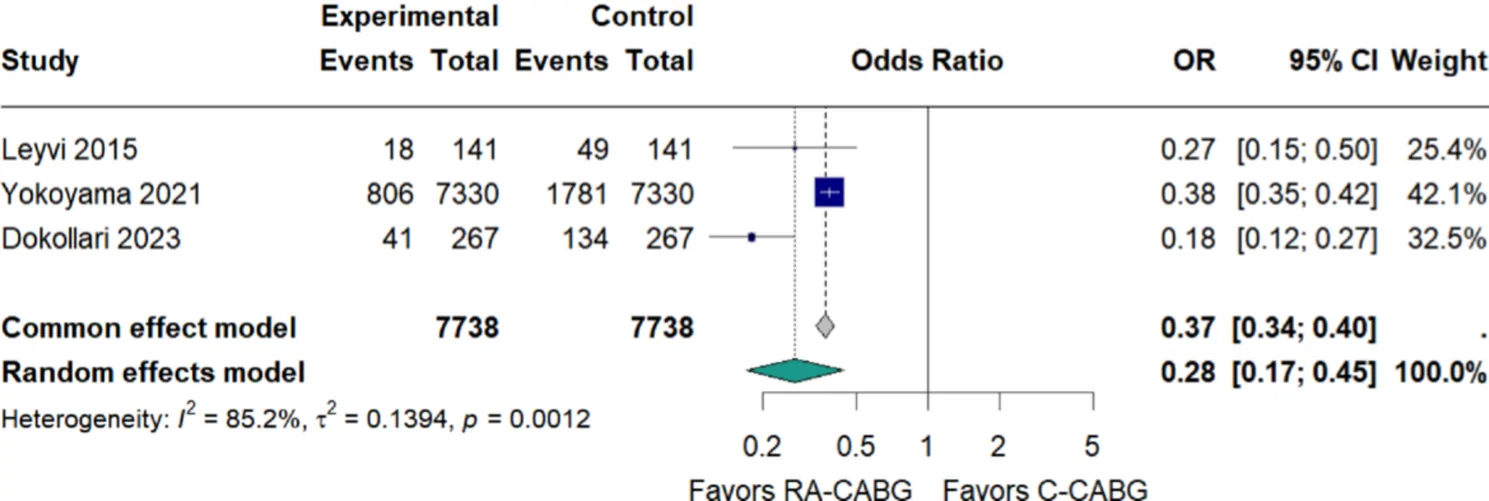
Forest Plot — Blood Transfusion

Three studies reported blood transfusion rates. RA-CABG was associated with substantially lower transfusion requirements compared to C-CABG (OR 0.28, 95% CI 0.17–0.45; *P* < 0.001, I² = 85%; 865/7,738 [11.2%] vs. 1,964/7,738 [25.4%]; Fig. 7). The high heterogeneity (I² = 85%) likely reflects differences in transfusion protocols and thresholds across institutions and time periods rather than true effect modification, as the direction of effect was consistent across all three studies.

#### Renal failure

Five studies reported postoperative renal failure. No significant difference was observed between RA-CABG and C-CABG (OR 0.73, 95% CI 0.46–1.16; *P* = 0.184, I² = 0%; 65/8,176 [0.79%] vs. 74/8,176 [0.90%]; Supplementary Figure S3).

#### Reoperation for bleeding

Four studies reported reoperation for bleeding. No significant difference was observed (OR 0.82, 95% CI 0.21–3.11; *P* = 0.767, I² = 54%; 13/846 [1.5%] vs. 15/846 [1.8%]; Supplementary Figure S4).

#### Sepsis

Three studies reported sepsis. No significant pooled difference was observed (OR 0.29, 95% CI 0.04–2.30; *P* = 0.240, I² = 60%; 81/7,809 [1.0%] vs. 147/7,809 [1.9%]; Supplementary Figure S5). This analysis was underpowered, with two of three studies having zero events in the robotic arm.

#### Deep Sternal Wound Infection (DSWI)

Three studies reported DSWI or mediastinitis. Zero events occurred in the RA-CABG group (0/508) compared to 6/508 in C-CABG. Due to sparse events and few contributing studies, a formal pooled analysis was not performed; this outcome is reported descriptively (Supplementary Figure S6).

#### Myocardial infarction

Two studies with non-zero events reported perioperative MI (Kofler 2017: 0/134 vs. 3/134; Poston 2008: 1/100 vs. 7/100). Due to the limited number of contributing studies, a formal meta-analysis was not performed, but the direction of effect favored RA-CABG in both studies (Supplementary Figure S7).

### Resource utilization outcomes

#### Hospital length of stay

**Fig. 8:**
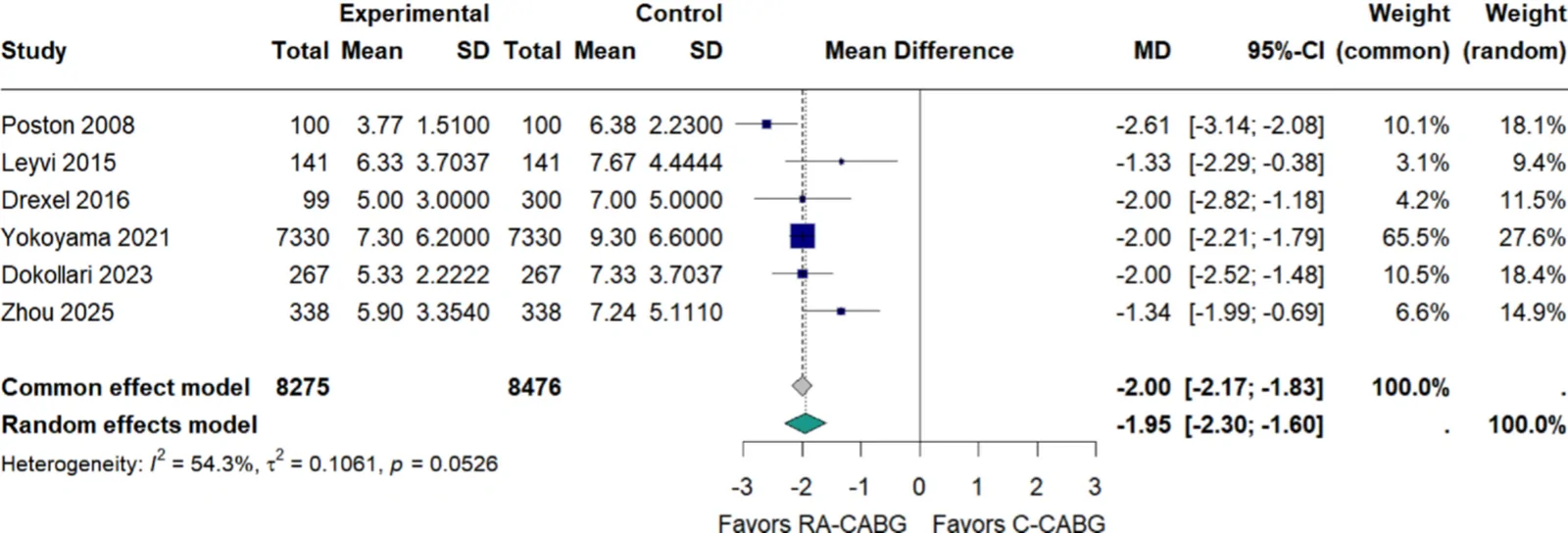
Forest Plot — Hospital Length of Stay (Mean Difference, Days)

Six studies reported hospital length of stay. RA-CABG was associated with significantly shorter hospital stay compared to C-CABG (MD − 1.95 days, 95% CI − 2.30 to − 1.60; *P* < 0.001, I² = 54%; Fig. 8). The moderate heterogeneity reflects variation in institutional discharge practices and patient populations across decades (study periods spanning 2001–2025). Despite this heterogeneity, the direction of effect was consistent across all six studies, and the pooled estimate remained statistically significant on leave-one-out analysis.

#### ICU length of stay

**Fig. 9:**
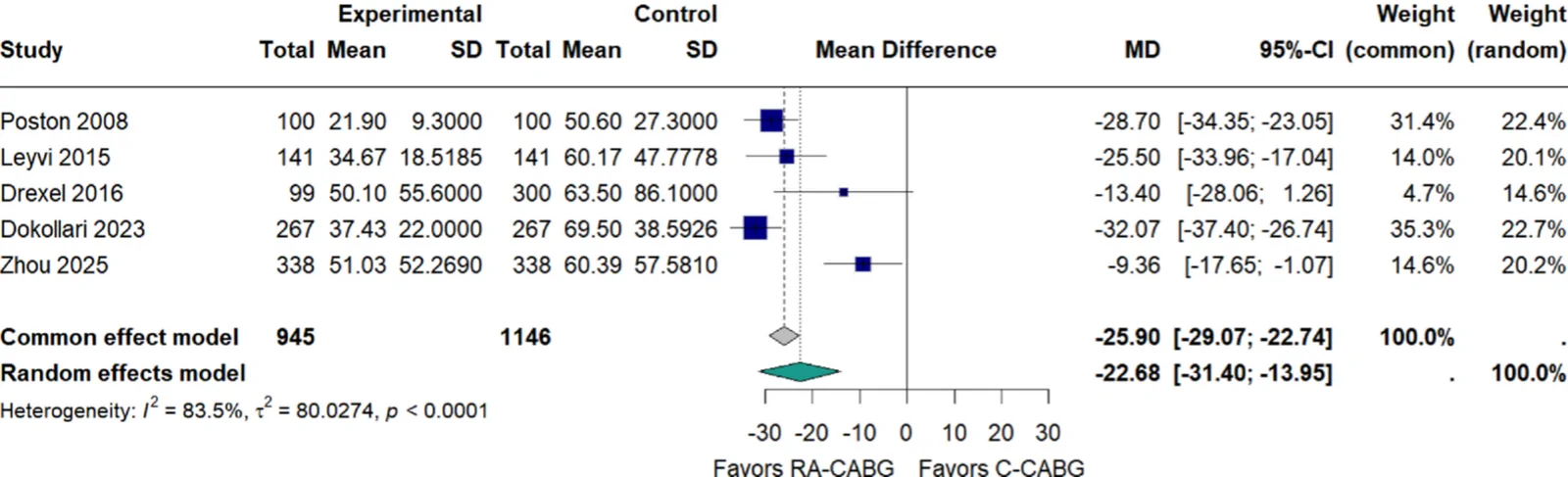
Forest Plot — ICU Length of Stay (Mean Difference, Hours)

Five studies reported ICU length of stay. RA-CABG was associated with significantly fewer ICU hours compared to C-CABG (MD − 22.7 h, 95% CI − 31.4 to − 14.0; *P* < 0.001, I² = 83%; Fig. 9). The high heterogeneity is attributable to variation in ICU management protocols across institutions and time periods, with Poston 2008 contributing the largest effect size (− 28.7 h) and Drexel 2016 contributing a smaller effect (− 13.4 h). Despite this heterogeneity, the random-effects pooled estimate and its confidence interval exclude zero, and all five studies reported shorter ICU stays in the RA-CABG group.

### Publication bias assessment

**Fig. 10:**
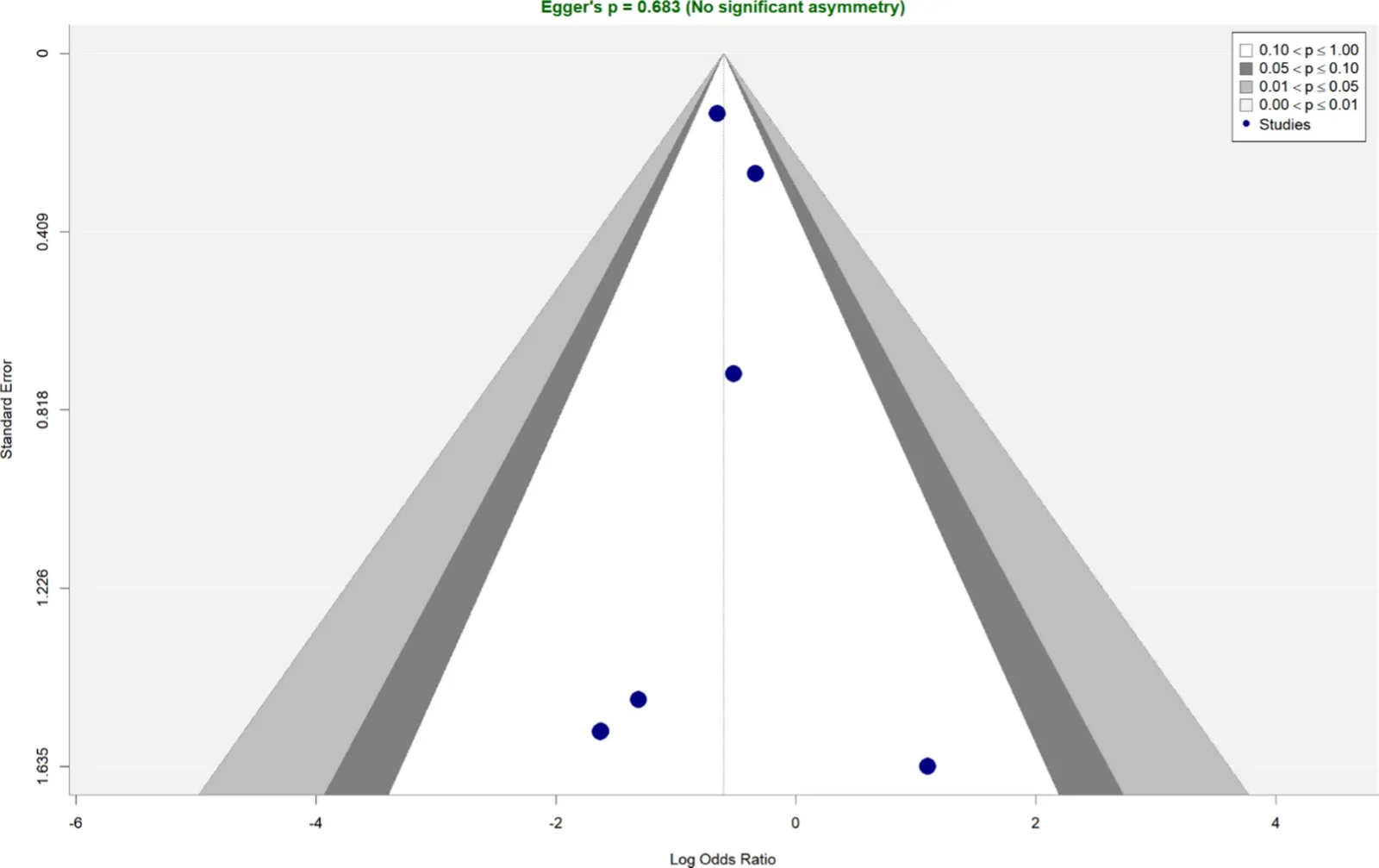
Funnel Plot — Mortality

Publication bias was assessed using funnel plots and Egger’s regression test for outcomes with a sufficient number of contributing studies. For mortality (8 studies), the funnel plot (Fig. 10) showed no marked asymmetry. Formal Egger’s testing was limited by the small number of studies per outcome (all < 10); results should be interpreted with caution. Funnel plots for stroke and other binary outcomes are presented in Supplementary Figures S10–S15.

### Sensitivity analyses

Leave-one-out sensitivity analyses were performed for all outcomes with three or more contributing studies. For mortality, sequential exclusion of each study produced point estimates ranging from OR 0.51 to 0.58, with all analyses remaining statistically significant (Supplementary Figure S2). Similar robustness was observed for atrial fibrillation, respiratory failure, and transfusion. These findings confirm that no single study disproportionately influenced the pooled results.

Pre-planned sensitivity analysis excluding Drexel 2016 (the only unmatched cohort, rated Serious risk of bias) did not materially alter the pooled mortality estimate (OR 0.56, 95% CI 0.43–0.72; Supplementary Figure S8) or stroke estimate (OR 0.77, 95% CI 0.57–1.04; Supplementary Figure S9), confirming that results are not dependent on the inclusion of this methodologically weaker study.

### Subgroup analyses

Prespecified subgroup analyses were performed according to publication year (< 2020 vs. ≥ 2020), geographic region (North America vs. Europe), and study size (< 500 vs. ≥ 500 patients) for outcomes with sufficient studies.

For mortality, subgroup analysis by publication year showed consistent associations in both earlier studies and recent studies, with no significant interaction (P-interaction > 0.10; Supplementary Figure S16). Geographic subgroup analysis demonstrated consistent mortality associations across regions without significant heterogeneity between subgroups. Subgroup analysis by study size showed similar associations in smaller and larger studies (Supplementary Figure S16).

For stroke, subgroup analyses by publication year, geographic region, and study size similarly demonstrated consistent non-significant trends without significant interactions (Supplementary Figure S17). Subgroup analyses for atrial fibrillation and renal failure showed consistent directions of effect across all subgroups (Supplementary Figures S18–S19).

### Meta-regression analyses

Meta-regression analyses using mixed-effects models were performed to explore publication year as a potential source of heterogeneity. For mortality, meta-regression found no significant association with publication year (Supplementary Figure S21), suggesting that the mortality association with RA-CABG has been consistent across the study period (2008–2025).

For stroke, meta-regression similarly showed no significant temporal trend (Supplementary Figure S22). For continuous outcomes including hospital length of stay and ICU stay, meta-regression did not identify publication year as a significant predictor of effect size, although the small number of contributing studies limits the power of these analyses.

### Trial sequential analysis

Trial sequential analysis was performed for all seven binary outcomes to determine whether the observed treatment effects had reached conclusive evidence thresholds (Supplementary Figures S32–S38). Results are summarized in Table 4.

**Table 5:** Trial Sequential Analysis Summary

Outcome	Studies (k)	Accrued *N*	RIS	Accrued/RIS (%)	Cumulative Z	Boundary Crossed?	Conclusion
Mortality	8	20,212	4,631	436%	−4.95	**Yes**	Conclusive: firm evidence of association
Stroke	7	17,019	7,314	233%	−1.81	**No**	Sufficient information; effect below threshold†
Atrial Fibrillation	4	1,809	239	757%	−5.41	**Yes**	Conclusive: firm evidence of association
Respiratory Failure	4	1,692	1,168	145%	−4.46	**Yes**	Conclusive: firm evidence of association
Blood Transfusion	3	15,476	275	5,627%	−5.28	**Yes**	Conclusive: firm evidence of association
Renal Failure	5	16,352	10,076	162%	−0.99	**No**	Sufficient information; effect below threshold†
Reoperation Bleeding	4	1,692	5,100	33%	−0.30	**No**	Inconclusive: insufficient information

RIS = Required Information Size, calculated assuming two-sided alpha = 0.05, power = 80%, and 40% relative risk reduction based on observed control event rate

† Accrued sample exceeds RIS, but the observed effect size is smaller than the assumed 40% RRR and does not reach the alpha-spending–adjusted significance threshold

For mortality, the cumulative Z-curve decisively crossed the TSA monitoring boundary (Z = − 4.95), with the accrued sample size (20,212 patients) exceeding the RIS (4,631 patients) by more than four-fold, confirming that the observed 45% relative reduction in mortality associated with RA-CABG represents conclusive evidence robust against random error (Supplementary Figure S32). Atrial fibrillation similarly demonstrated conclusive evidence (Z = − 5.41; RIS = 239; accrued = 1,809 patients; Supplementary Figure S34), with the Z-curve crossing the monitoring boundary after the inclusion of only four studies. Respiratory failure achieved conclusive status (Z = − 4.46; RIS = 1,168; accrued = 1,692 patients; Supplementary Figure S35), with the accrued information exceeding the RIS by 45%. Blood transfusion also reached conclusive evidence (Z = − 5.28; RIS = 275; accrued = 15,476 patients; Supplementary Figure S36), reflecting the large and consistent effect size across three studies (pooled OR 0.28).

For stroke, although the accrued sample size (17,019 patients) exceeded the RIS (7,314 patients) by more than two-fold, the cumulative Z-curve (Z = − 1.81) did not cross the O’Brien–Fleming monitoring boundary (Supplementary Figure S33). This indicates that the accumulated evidence is sufficient in quantity, but the observed effect size (OR 0.76, representing a 24% relative reduction) is too small to achieve statistical significance at the alpha-spending–adjusted threshold. Unlike outcomes where the RIS has not been reached, this result suggests that the true effect of RA-CABG on stroke risk—if present—is likely smaller than the 40% RRR assumed in the TSA calculation, and that substantially larger studies or a genuinely larger effect would be needed to reach a conclusive result. Renal failure showed a similar pattern (Z = − 0.99; RIS = 10,076; accrued = 16,352 patients; Supplementary Figure S37), where the accrued information exceeded the RIS but the Z-curve did not cross the monitoring boundary, confirming the non-significant pooled result (OR 0.73, *P* = 0.184). For reoperation for bleeding, the TSA was notably underpowered (Z = − 0.30; RIS = 5,100; accrued = 1,692 patients, representing only 33% of the RIS; Supplementary Figure S38), precluding definitive conclusions for this outcome.

In summary, TSA confirmed conclusive evidence for four of seven binary outcomes (mortality, atrial fibrillation, respiratory failure, and blood transfusion), strengthening confidence that these clinically significant associations are not attributable to random error or the cumulative effects of multiple hypothesis tests. Two outcomes (stroke and renal failure) had sufficient accrued information but effect sizes below the monitoring threshold, while one outcome (reoperation for bleeding) requires further accumulation of evidence—ideally from prospective randomized controlled trials—before definitive conclusions can be drawn.

## Discussion

This systematic review and meta-analysis of nine observational studies comprising 39,918 patients constitutes, to our knowledge, the most comprehensive comparative analysis of robotic-assisted versus conventional coronary artery bypass grafting published to date. The principal findings are fourfold: first, RA-CABG was associated with a 45% relative reduction in short-term mortality (OR 0.55, 95% CI 0.43–0.69; *P* < 0.001; I² = 0%); second, RA-CABG was associated with significantly lower rates of atrial fibrillation (OR 0.53), respiratory failure (OR 0.31), and blood transfusion requirements (OR 0.28); third, hospital and ICU lengths of stay were significantly shorter; and fourth, trial sequential analysis confirmed that the evidence for mortality, atrial fibrillation, respiratory failure, and transfusion has crossed the threshold for conclusive statistical reliability. Notably, heterogeneity was negligible (I² = 0%) for most binary outcomes, and results remained robust across leave-one-out sensitivity analyses, subgroup analyses, and pre-planned exclusion of the single unmatched cohort.

These findings complement and substantially extend the existing evidence base. Yokoyama et al., in their analysis of the National Inpatient Sample, reported that robotic CABG was associated with lower rates of in-hospital mortality, acute kidney injury, transfusion, and postoperative hemorrhage compared to nonrobotic CABG [18]. Similarly, Hammal et al. conducted a systematic review and meta-analysis of comparative studies and concluded that findings appear promising and suggest that robot-assisted coronary bypass surgery may offer some benefits to patients; however, in the absence of randomized controlled trials, they advised that results should be interpreted cautiously [12]. While these prior investigations provided meaningful preliminary evidence, they were constrained by smaller pooled samples and limited outcome breadth. Our analysis, incorporating additional contemporary studies and a total patient population exceeding 39,000, provides substantially greater statistical power and narrower confidence intervals.

An important methodological consideration is the potential for case-selection bias. The RA-CABG cohorts consistently underwent fewer grafts (range 1.1–1.9) compared to C-CABG (2.5–3.1), reflecting the current predominant application of robotic techniques to single-vessel or limited multivessel disease. This lower baseline complexity may partially bias early outcomes in favor of RA-CABG, as patients with less extensive coronary disease and fewer comorbidities may inherently carry lower perioperative risk regardless of surgical approach. Although seven of nine studies employed propensity score matching or entropy balancing to adjust for measured confounders, these methods cannot account for unmeasured confounders or for the fundamental difference in revascularization completeness between groups. The observed associations should therefore be interpreted within the context of current RA-CABG patient selection practices rather than as definitive evidence of superiority across all CABG populations.

An important related consideration is the role of hybrid coronary revascularization. RA-CABG is frequently performed as a robotic LIMA-to-LAD anastomosis combined with PCI to non-LAD territories, providing functionally complete revascularization through a staged or simultaneous hybrid approach. However, none of the nine included studies systematically reported hybrid revascularization rates, completeness of revascularization indices, or PCI co-intervention data. This represents a significant gap in the current literature, as the apparent difference in graft numbers between RA-CABG and C-CABG cohorts may overstate the true difference in revascularization completeness if hybrid strategies were employed but not reported. Future comparative studies should mandate systematic reporting of hybrid PCI rates and long-term completeness of revascularization to enable proper assessment of the overall revascularization strategy.

The 45% relative reduction in mortality observed in association with RA-CABG is particularly noteworthy and warrants careful mechanistic consideration. This observed survival association is unlikely to derive from a single factor; rather, it appears to reflect the cumulative aggregate effect of simultaneous reductions across multiple complication domains. Our analysis demonstrates that RA-CABG patients experienced lower rates of atrial fibrillation, respiratory failure, blood transfusion, and a non-significant trend toward fewer strokes—each of which independently contributes to perioperative mortality risk. The convergence of these protective effects, combined with the avoidance of sternotomy and the associated preservation of chest wall integrity, likely creates a synergistic reduction in the overall burden of postoperative morbidity that may contribute to the observed lower mortality. However, it must be acknowledged that the lower procedural complexity of RA-CABG cases (fewer grafts, predominantly single-vessel disease) and inherent case-selection bias may account for a substantial proportion of the observed mortality difference, independent of the surgical approach itself.

Although the pooled analysis for stroke did not reach conventional statistical significance (OR 0.76, 95% CI 0.57–1.02; *P* = 0.071), the 24% relative reduction in favor of RA-CABG is mechanistically plausible. Kapetanakis et al. demonstrated that cerebral embolization of atherosclerotic plaque debris caused by aortic manipulation during conventional CABG is a major mechanism of postoperative stroke, and that the incidence of stroke increases with increased levels of aortic manipulation [19]. Robotic-assisted CABG, particularly when performed as TECAB, inherently eliminates direct aortic instrumentation. The clinical feasibility of this approach is well established: Balkhy et al. reported outcomes of 570 consecutive off-pump TECAB procedures with an observed mortality of 0.6%, a completion rate of 98.8%, and graft patency exceeding 95% [20]. Furthermore, Liu 2025 reported a significant long-term reduction in cerebrovascular events (HR 0.771, 95% CI 0.600–0.991; *P* = 0.042), suggesting that the potential neuroprotective advantages may become more apparent over extended observation periods.

The significant reduction in postoperative atrial fibrillation (OR 0.53, 95% CI 0.42–0.66; *P* < 0.001) is among the most consistent findings, with all four contributing studies demonstrating the same direction of effect and no heterogeneity (I² = 0%). Dobrev et al. described the underlying mechanisms as including inflammation, sympathetic activation, and cardiac ischaemia, which combine to trigger atrial fibrillation, often in the presence of pre-existing factors making the atria vulnerable [21]. By avoiding median sternotomy and minimizing pericardial dissection, robotic approaches inherently attenuate several of these triggers. Nevertheless, the contribution of patient selection—with RA-CABG patients potentially having less extensive disease and lower inflammatory burden—cannot be excluded.

The 69% relative reduction in respiratory failure (OR 0.31, 95% CI 0.18–0.52; *P* < 0.001) represents perhaps the most striking individual outcome association. Enhanced recovery protocols in cardiac surgery emphasize the importance of minimizing surgical trauma to optimize postoperative respiratory and overall outcomes [5]. Robotic-assisted approaches preserve the structural continuity of the sternum entirely, utilizing only small intercostal port incisions that minimally affect respiratory mechanics. However, the lower complexity of robotic cases may also contribute to this finding, as patients undergoing single-vessel grafting may have shorter operative times and less hemodynamic perturbation.

The association between RA-CABG and substantially lower blood transfusion requirements (OR 0.28, 95% CI 0.17–0.45; *P* < 0.001) carries implications beyond immediate blood product utilization. Murphy et al. demonstrated that red blood cell transfusion in patients having cardiac surgery is strongly associated with both infection and ischemic postoperative morbidity, increased early and late mortality, and higher hospital costs [22]. The association between RA-CABG and lower transfusion requirements may reflect reduced tissue disruption inherent to port-based approaches, though differences in procedural complexity and case selection may also contribute.

The finding of zero DSWI events across robotic cohorts (0/508), while biologically plausible given the absence of sternotomy, warrants cautious interpretation. DSWI definitions varied across studies and did not uniformly follow Society of Thoracic Surgeons (STS) criteria; some studies used institutional definitions of mediastinitis or deep infection. Furthermore, retrospective datasets may underreport wound complications, particularly when follow-up is limited to the index hospitalization. Although the direction of effect is consistent with the biological rationale of sternotomy avoidance, this outcome should be considered hypothesis-generating rather than conclusive, and prospective studies with standardized STS definitions are needed to confirm this finding.

While binary outcomes demonstrated reassuring consistency (I² = 0% for most), continuous outcomes exhibited substantial to considerable heterogeneity (I² = 54% for hospital LOS; I² = 83% for ICU LOS). This heterogeneity likely reflects real clinical variation across institutions and eras rather than statistical artifact: discharge criteria, ICU transfer protocols, and postoperative care pathways differ substantially between centers and have evolved over the 17-year study period (2001–2025). Although the random-effects model accommodates this heterogeneity, the pooled estimates for continuous outcomes should be interpreted as representing average treatment effects across diverse clinical contexts rather than precise point estimates applicable to any single institution.

The significant reductions in hospital length of stay (MD − 1.95 days; *P* < 0.001) and ICU stay (MD − 22.7 h; *P* < 0.001) carry important economic implications. Dokollari et al. demonstrated that total hospital costs were substantially lower in the robotic-assisted CABG group ($18,726 per patient) compared with conventional CABG ($35,580 per patient), with the shorter hospital length of stay identified as the primary driver of cost reduction [23].

A particularly important methodological contribution of this analysis is the systematic application of trial sequential analysis across all binary outcomes. Our TSA confirmed that conclusive evidence thresholds have been crossed for four of seven binary outcomes, indicating that the observed associations are statistically firm and unlikely to be overturned by future studies of similar size. Importantly, while TSA strengthens confidence that these observed associations are not attributable to random error or the cumulative effects of multiple hypothesis testing, it does not address systematic biases inherent in observational studies—including confounding, selection bias, and measurement error. The conclusive TSA results should therefore be interpreted as confirming the statistical reliability of the observed associations within the limitations of the available observational evidence, rather than as establishing causal superiority. Conversely, stroke, renal failure, and reoperation for bleeding remain inconclusive, delineating specific areas where additional evidence is required.

An important consideration in interpreting these results is the concentration of robotic CABG experience within high-volume specialty centers. Nearly all included studies originated from institutions with established robotic cardiac surgery programs, where surgeon expertise, institutional protocols, and patient selection have been refined over years of cumulative experience. Surgeon and center volume are well-recognized determinants of outcomes in cardiac surgery, and the results of this meta-analysis may not be directly generalizable to lower-volume or emerging robotic programs, where learning-curve effects could attenuate or reverse the observed associations. Jonsson et al. reported that the learning curve to achieve mastery in robotic CABG comprises approximately 250 to 500 cases [24], reinforcing the need for structured training programs and volume thresholds before outcomes comparable to those reported here can be expected.

The included studies span a period of rapid evolution in robotic cardiac surgery technology (2008–2025). During this interval, robotic platforms, instrumentation, and procedural standardization underwent substantial improvements, potentially influencing reported outcomes. Earlier studies may reflect higher operative times and complication rates associated with first-generation systems and early institutional experience, while more recent studies may benefit from refined techniques, improved patient selection algorithms, and enhanced recovery protocols. Although our meta-regression found no significant association between publication year and effect size for mortality or stroke, the limited number of studies constrains the power of this analysis to detect temporal trends.

As the field evolves, appropriate safeguards in the adoption of robotic surgery have been highlighted as essential for ensuring patient safety during the dissemination of this technology [25]. This includes structured credentialing pathways, minimum case-volume requirements, and standardized reporting of patient-reported outcomes to ensure that the observed associations in high-volume centers are not compromised during the expansion of robotic CABG to broader surgical communities.

### Limitations

Several limitations merit consideration. First, all nine included studies were observational, and despite the use of propensity score matching or entropy balancing in eight of nine studies, residual confounding cannot be excluded. In particular, case-selection bias—with RA-CABG preferentially applied to patients with single-vessel disease and lower comorbidity burden—represents a systematic source of confounding that propensity matching on measured covariates alone cannot fully address. Second, long-term follow-up data were insufficiently reported, limiting assessment of durability. Only Liu 2025 reported outcomes beyond 30 days (median 49 months), and no study reported long-term graft patency, functional status, or quality of life—outcomes that are critical to the overall value assessment of robotic CABG. Third, the number of grafts was consistently lower in RA-CABG cohorts (range 1.1–1.9) compared to C-CABG cohorts (range 2.5–3.1), reflecting the current predominant application of RA-CABG to patients with single-vessel or limited multivessel coronary disease. This imbalance is central to interpretation, as robotic cohorts likely represented a more selected patient population with less extensive coronary disease and potentially fewer comorbidities, which may independently confer lower perioperative risk regardless of surgical approach. Propensity score matching on measured covariates cannot fully account for this fundamental difference in disease complexity and revascularization strategy. Fourth, none of the included studies reported hybrid revascularization rates (robotic LIMA-to-LAD combined with PCI to non-LAD territories), precluding assessment of completeness of revascularization. Fifth, temporal variation across the 17-year study period (2008–2025) introduces potential confounding from concurrent advances. Sixth, the concentration of evidence from high-volume specialty centers limits generalizability to emerging programs. Seventh, DSWI definitions were not standardized across studies, and the zero-event finding should be considered preliminary. Eighth, Egger’s regression test has limited statistical power when fewer than 10 studies are included, as is the case for all outcomes in this meta-analysis, and the results of publication bias assessment should therefore be interpreted with particular caution. Finally, three outcomes remain inconclusive by TSA, and no randomized controlled trials are available.

## Conclusions

This meta-analysis of 39,918 patients from nine observational studies suggests that robotic-assisted CABG is associated with significantly lower mortality, atrial fibrillation, respiratory failure, blood transfusion requirements, hospital length of stay, and ICU stay compared with conventional CABG. Trial sequential analysis confirmed conclusive evidence for four of seven binary outcomes. However, these findings must be interpreted in the context of observational data, potential case-selection bias favoring lower-complexity patients in RA-CABG cohorts, and the concentration of evidence from high-volume centers. Future research should prioritize multicenter randomized controlled trials with long-term follow-up and standardized reporting of graft patency, hybrid revascularization rates, and functional outcomes.

## Supplementary Information

Below is the link to the electronic supplementary material.

Supplementary Material 1

Supplementary Material 2

Supplementary Material 3

## Data Availability

All data generated or analyzed during this study are included in this published article and its supplementary information files.
