# Supplementary material for: Robotic-assisted versus conventional coronary artery bypass grafting: a systematic review, meta-analysis, and trial sequential analysis of 39,918 patients
Source: J Cardiothorac Surg. 2026 Oct 12;21:763. doi: 10.1186/s13019-026-04684-8 (PMC13660123; doi:10.1186/s13019-026-04684-8)
Supplement: Supplementary file 1 [file 13019_2026_4684_MOESM1_ESM.docx]

**SUPPLEMENTARY MATERIAL**

**Robotic-Assisted versus Conventional Coronary Artery Bypass Grafting: A Systematic Review and Meta-Analysis**

**Supplementary Table S1. Comprehensive Search Strategy**

**PubMed/MEDLINE:** *((robotic[tiab] OR "robot-assisted"[tiab] OR "da Vinci"[tiab] OR TECAB[tiab] OR RMIDCAB[tiab] OR telemanipulation[tiab]) AND ("coronary artery bypass"[tiab] OR CABG[tiab] OR "coronary revascularization"[tiab])) AND (comparative[tiab] OR matched[tiab] OR cohort[tiab] OR retrospective[tiab] OR prospective[tiab])*

**Embase:** *(robotic OR robot-assisted OR da:Vinci OR TECAB OR RMIDCAB) AND (coronary:artery:bypass:graft* OR CABG) AND (comparative OR matched OR cohort)*

**Cochrane CENTRAL:** *(robotic OR robot-assisted) AND (coronary artery bypass OR CABG)*

**Web of Science:** *TS=(robotic CABG OR robotic coronary artery bypass OR TECAB OR RMIDCAB)*

Search date: January 31, 2026. Language: No restriction. Date limits: None.

**Supplementary Table S2. PRISMA 2020 Checklist**

| **Section/Topic** | **Item No.** | **Checklist Item** | **Location** | **✓/NA** |
| --- | --- | --- | --- | --- |
| Title | 1 | Identify the report as a systematic review. | Title page | ✓ |
| Abstract | 2 | Structured abstract with background, methods, results, conclusions. | Abstract | ✓ |
| Introduction – Rationale | 3 | Rationale for the review. | Section 1 | ✓ |
| Introduction – Objectives | 4 | Explicit objectives and PICO. | Section 1 | ✓ |
| Methods – Eligibility | 5 | Eligibility criteria specified. | Section 2.3 | ✓ |
| Methods – Sources | 6 | Information sources and search dates. | Section 2.2 | ✓ |
| Methods – Search | 7 | Full search strategy for one database. | Supp. Table S1 | ✓ |
| Methods – Selection | 8 | Selection process. | Section 2.4 | ✓ |
| Methods – Data collection | 9 | Data collection process. | Section 2.4 | ✓ |
| Methods – Data items | 10a | All outcomes sought. | Section 2.5 | ✓ |
| Methods – Risk of bias | 11 | Risk of bias assessment. | Section 2.6 | ✓ |
| Methods – Statistics | 13 | Statistical synthesis methods. | Section 2.7 | ✓ |
| Results – Selection | 16 | Study selection results with PRISMA diagram. | Section 3.1 + Fig S1 | ✓ |
| Results – Study characteristics | 17 | Characteristics of studies. | Table 1 | ✓ |
| Results – Risk of bias | 18 | Risk of bias assessments. | Table 3 + Figs S2–S3 | ✓ |
| Results – Synthesis | 20a | Pooled results for all outcomes. | Table 2 + Figs 1–4 | ✓ |
| Discussion – Limitations | 27 | Discussion of limitations. | Section 4.8 | ✓ |
| Discussion – Conclusions | 28 | General interpretation. | Section 5 | ✓ |
| Registration – Registration | 24a | PROSPERO registration. | Section 2.1 | ✓ |

**Supplementary Table S3. Verified Event Counts by Outcome**

*All values verified line-by-line against source full-texts. NA = outcome not reported in study. † = Sensitivity analysis only (Drexel 2016, unmatched).*

| **Study** | **Mortality R/N** | **Mortality C/N** | **Stroke R/N** | **Stroke C/N** | **AF R/N** | **AF C/N** | **Renal R/N** | **Renal C/N** | **Resp R/N** | **Resp C/N** | **Reop R/N** | **Reop C/N** | **LOS R (days)** | **LOS C (days)** | **ICU R (hrs)** | **ICU C (hrs)** |
| --- | --- | --- | --- | --- | --- | --- | --- | --- | --- | --- | --- | --- | --- | --- | --- | --- |
| **Kofler 2017** | 0/134 | 2/134 | 0/134 | 1/134 | NA | NA | NA | NA | NA | NA | NA | NA | NA | NA | NA | NA |
| **Dokollari 2023** | 1/267 | 0/267 | 2/267 | 2/267 | 47/267 | 81/267 | 1/267 | 5/267 | 4/267 | 15/267 | 2/267 | 1/267 | 5 (IQR 4–7) | 7 (IQR 5–10) | 31.2 (IQR) | 65.0 (IQR) |
| **Yokoyama 2021** | 81/7330 | 154/7330 | 73/7330 | 95/7330 | NA | NA | 59/7330† | 59/7330† | NA | NA | NA | NA | 7.3±6.2 | 9.3±6.6 | NA | NA |
| **Liu 2025** | 23/1796 | 32/1796 | HR 0.77‡ | — | NA | NA | NA | NA | NA | NA | NA | NA | NA | NA | NA | NA |
| **Zhou 2025** | 3/338 | 5/338 | 4/338 | 2/338 | 64/338 | 107/338 | 1/338 | 4/338 | 8/338 | 28/338 | 8/338 | 3/338 | 5.90±3.35 | 7.24±5.11 | 51.0±52.3 | 60.4±57.6 |
| **Verma 2025** | AOR 0.35§ | — | — | — | NA | NA | NA | NA | NA | NA | NA | NA | 4 (IQR 3–5) | 5 (IQR 4–7) | NA | NA |
| **Drexel 2016†** | 0/99 | 5/300 | 0/99 | 3/300 | 18/99 | 75/300 | NA | NA | NA | NA | NA | NA | 5±3 | 7±5 | 50.1±55.6 | 63.5±86.1 |
| **Poston 2008** | 0/100 | 2/100 | 0/100 | 2/100 | 12/100 | 20/100 | 3/100 | 5/100 | 6/100 | 12/100 | 1/100 | 6/100 | 3.77±1.51 | 6.38±2.23 | 21.9±9.3 | 50.6±27.3 |
| **Leyvi 2015** | 0/141 | 2/141 | 0/141 | 2/141 | NA | NA | 1/141 | 1/141 | 2/141 | 7/141 | 2/141 | 5/141 | 6 (IQR 4–9) | 7 (IQR 5–11) | 31 (IQR) | 52 (IQR) |

*R = Robotic; C = Conventional; N = denominator; AF = Atrial Fibrillation; Resp = Respiratory Failure/Prolonged Ventilation; Reop = Reoperation for Bleeding; LOS = Length of Stay; ICU = ICU Stay; IQR = Interquartile Range; † Drexel 2016: unmatched comparison, sensitivity analysis only. ‡ Liu 2025 stroke: Kaplan-Meier HR 0.771 (0.600–0.991); not pooled in binary forest plot. § Verma 2025 mortality: adjusted OR via entropy balancing; not pooled as raw counts.*
