# Supplementary material for: Robotic-assisted versus conventional coronary artery bypass grafting: a systematic review, meta-analysis, and trial sequential analysis of 39,918 patients
Source: J Cardiothorac Surg. 2026 Oct 12;21:763. doi: 10.1186/s13019-026-04684-8 (PMC13660123; doi:10.1186/s13019-026-04684-8)
Supplement: Supplementary file 2 [file 13019_2026_4684_MOESM2_ESM.docx]

**Supplementary Figure S1. ROBINS-I Summary Bar Chart**

**Supplementary Figure S2. Leave-One-Out — Mortality**

**Supplementary Figure S3. Forest Plot — Renal Failure**

**Supplementary Figure S4. Forest Plot — Reoperation for Bleeding**

**Supplementary Figure S5. Forest Plot — Sepsis**

**Supplementary Figure S6. Forest Plot — Deep Sternal Wound Infection**

**Supplementary Figure S7. Forest Plot — Myocardial Infarction**

**Supplementary Figure S8. Sensitivity — Mortality (Excluding Drexel 2016)**

**Supplementary Figure S9. Sensitivity — Stroke (Excluding Drexel 2016)**

**Supplementary Figure S10. Funnel Plot — Stroke**

**Supplementary Figure S11. Funnel Plot — Atrial Fibrillation**

**Supplementary Figure S12. Funnel Plot — Renal Failure**

**Supplementary Figure S13. Funnel Plot — Respiratory Failure**

**Supplementary Figure S14. Funnel Plot — Reoperation**

**Supplementary Figure S15. Funnel Plot — Transfusion**

**Supplementary Figure S16. Subgroup Analysis — Mortality**

**Supplementary Figure S17. Subgroup Analysis — Stroke**

**Supplementary Figure S18. Subgroup Analysis — Atrial Fibrillation**

**Supplementary Figure S19. Subgroup Analysis — Renal Failure**

**Supplementary Figure S20. Subgroup Analysis — Respiratory Failure**

**Supplementary Figure S21. Meta-Regression — Mortality by Year**

**Supplementary Figure S22. Meta-Regression — Stroke by Year**

**Supplementary Figure S23. Meta-Regression — Atrial Fibrillation by Year**

**Supplementary Figure S24. Meta-Regression — Renal Failure by Year**

**Supplementary Figure S25. Meta-Regression — Respiratory Failure by Year**

**Supplementary Figure S26. Leave-One-Out — Stroke**

**Supplementary Figure S27. Leave-One-Out — Atrial Fibrillation**

**Supplementary Figure S28. Leave-One-Out — Renal Failure**

**Supplementary Figure S29. Leave-One-Out — Respiratory Failure**

**Supplementary Figure S30. Leave-One-Out — Reoperation**

**Supplementary Figure S31. Leave-One-Out — Transfusion**

**Supplementary Figure S32. Trial Sequential Analysis — Mortality (RA-CABG vs C-CABG)**

**Supplementary Figure S33. Trial Sequential Analysis — Stroke (RA-CABG vs C-CABG)**

**Supplementary Figure S34. Trial Sequential Analysis — Atrial Fibrillation (RA-CABG vs C-CABG)**

**Supplementary Figure S35. Trial Sequential Analysis — Respiratory Failure (RA-CABG vs C-CABG)**

**Supplementary Figure S36. Trial Sequential Analysis — Blood Transfusion (RA-CABG vs C-CABG)**

**Supplementary Figure S37. Trial Sequential Analysis — Renal Failure (RA-CABG vs C-CABG)**

**Supplementary Figure S38. Trial Sequential Analysis — Reoperation for Bleeding (RA-CABG vs C-CABG)**
