# Supplementary material for: Robotic-assisted versus conventional coronary artery bypass grafting: a systematic review, meta-analysis, and trial sequential analysis of 39,918 patients
Source: J Cardiothorac Surg. 2026 Oct 12;21:763. doi: 10.1186/s13019-026-04684-8 (PMC13660123; doi:10.1186/s13019-026-04684-8)
Supplement: Supplementary file 3 [file 13019_2026_4684_MOESM3_ESM.docx]

**Supplementary References: Included Studies**

The following nine studies were included in this systematic review and meta-analysis. Eight studies are indexed on PubMed; one study (Drexel 2016) is published in a non-PubMed-indexed journal. All references were managed using Zotero citation management software and verified against PubMed/CrossRef.

1. Poston RS, Tran R, Collins M, et al. Comparison of economic and patient outcomes with minimally invasive versus traditional off-pump CABG techniques. Ann Surg. 2008;248(4):638-46. doi:10.1097/SLA.0b013e31818a15b5. PMID: 18936577.

2. Leyvi G, Schechter CB, Sehgal S, et al. Comparison of Index Hospitalization Costs Between Robotic CABG and Conventional CABG. J Cardiothorac Vasc Anesth. 2016;30(1):12-8. doi:10.1053/j.jvca.2015.07.031. PMID: 26597467.

3. Drexel TS, Reyes M, Andrei AC, Raff GW. Hybrid and Robotic-Assisted Coronary Artery Revascularization Compared to Conventional CABG. J Cardiol Cardiovasc Ther. 2016;1(1):555562.

4. Kofler M, Schachner T, Reinstadler SJ, et al. Comparative Analysis of TECAB and MIDCAB for Revascularization of Anterior Wall. Innovations. 2017;12(3):207-213. doi:10.1097/IMI.0000000000000378. PMID: 28542076.

5. Yokoyama Y, Kuno T, Malik A, Briasoulis A. Outcomes of robotic vs nonrobotic CABG. J Card Surg. 2021;36(9):3187-3192. doi:10.1111/jocs.15710. PMID: 34091953.

6. Dokollari A, Sicouri S, Prendergrast G, et al. Robotic vs Full-Sternotomy CABG: Hospital Costs. Am J Cardiol. 2024;213:12-19. doi:10.1016/j.amjcard.2023.10.083. PMID: 38012991.

7. Liu CH, Wu YS, Yu CL, Wang CC, Wei HJ. Comparative long-term effectiveness of robotic vs non-robotic CABG: TriNetX analysis. J Robot Surg. 2025;19(1):726. doi:10.1007/s11701-025-02905-1. PMID: 41165918.

8. Verma A, Kim JJ, Sakowitz S, et al. Association of Robotic Assistance With Short-term Outcomes After CABG. Ann Thorac Surg Short Rep. 2025;3(3):603-608. doi:10.1016/j.atssr.2025.03.007. PMID: 41163903.

9. Zhou Z, Gleboff A, Dilip K, et al. Propensity-Matched Outcomes of Multivessel Robotic vs Conventional Coronary Grafting. Ann Thorac Surg Short Rep. 2025;3(3):598-602. doi:10.1016/j.atssr.2025.01.001. PMID: 41163896.

**Verification Summary**

| **#** | **Study** | **PMID** | **Journal** | **DOI Verified** |
| --- | --- | --- | --- | --- |
| 1 | Poston 2008 | 18936577 | Ann Surg | Verified |
| 2 | Leyvi 2015 | 26597467 | J Cardiothorac Vasc Anesth | Verified |
| 3 | Drexel 2016 | N/A | J Cardiol Cardiovasc Ther | Publisher PDF |
| 4 | Kofler 2017 | 28542076 | Innovations | Verified |
| 5 | Yokoyama 2021 | 34091953 | J Card Surg | Verified |
| 6 | Dokollari 2023 | 38012991 | Am J Cardiol | Verified |
| 7 | Liu 2025 | 41165918 | J Robot Surg | Corrected R2 |
| 8 | Verma 2025 | 41163903 | Ann Thorac Surg Short Rep | Corrected R2 |
| 9 | Zhou 2025 | 41163896 | Ann Thorac Surg Short Rep | Corrected R2 |
|  | **Total: 9 studies** | **8/9 indexed** |  | **9/9 verified** |
